# Clinical features and outcomes of MEN2A with *RET* codon 634 pathogenic variants

**DOI:** 10.1210/jendso/bvag254

**Published:** 2026-10-31

**Authors:** Sarah Hamidi, Steven G Waguespack, Naifa L Busaidy, Paul H Graham, Mouhammed A Habra, Nancy D Perrier, Steven I Sherman, Catherine B Skefos, Julie Ann Sosa, Anita K Ying, Mark E Zafereo, Elizabeth G Grubbs, Mimi I Hu

**Affiliations:** Department of Endocrine Neoplasia and Hormonal Disorders, The University of Texas MD Anderson Cancer Center, Houston, TX 77030, USA; Department of Endocrine Neoplasia and Hormonal Disorders, The University of Texas MD Anderson Cancer Center, Houston, TX 77030, USA; Department of Endocrine Neoplasia and Hormonal Disorders, The University of Texas MD Anderson Cancer Center, Houston, TX 77030, USA; Department of Surgical Oncology, The University of Texas MD Anderson Cancer Center, Houston, TX 77030, USA; Department of Endocrine Neoplasia and Hormonal Disorders, The University of Texas MD Anderson Cancer Center, Houston, TX 77030, USA; Section of Endocrinology, King Faisal Specialist Hospital and Research Centre, Riyadh 11211, Saudi Arabia; Department of Surgical Oncology, The University of Texas MD Anderson Cancer Center, Houston, TX 77030, USA; Department of Endocrine Neoplasia and Hormonal Disorders, The University of Texas MD Anderson Cancer Center, Houston, TX 77030, USA; Department of Endocrine Neoplasia and Hormonal Disorders, The University of Texas MD Anderson Cancer Center, Houston, TX 77030, USA; Clinical Cancer Genetics Program, The University of Texas MD Anderson Cancer Center, Houston, TX 77030, USA; Department of Surgery, University of California San Francisco, San Francisco, CA 94143, USA; Department of Endocrine Neoplasia and Hormonal Disorders, The University of Texas MD Anderson Cancer Center, Houston, TX 77030, USA; Department of Head and Neck Surgery, The University of Texas MD Anderson Cancer Center, Houston, TX 77030, USA; Department of Surgical Oncology, The University of Texas MD Anderson Cancer Center, Houston, TX 77030, USA; Department of Endocrine Neoplasia and Hormonal Disorders, The University of Texas MD Anderson Cancer Center, Houston, TX 77030, USA

**Keywords:** multiple endocrine neoplasia type 2, medullary thyroid cancer, pheochromocytoma, hyperparathyroidism, survival, genotype–phenotype correlation

## Abstract

**Background:**

Germline *RET* codon 634 pathogenic variants (PVs) are at high-risk for early-onset medullary thyroid carcinoma (MTC), but prior reports suggest that *RET* p.C634R PV may confer a more aggressive phenotype.

**Methods:**

We compared MEN2A phenotype and MTC outcomes with germline *RET* p.C634R vs other *RET* p.C634X PVs. We conducted a retrospective single-center cohort study of patients harboring a germline *RET* p.C634 PV, enrolled in the MTC Collaborative Registry (MTCCoRe) and seen at least once between May 2012 and May 2024.

**Results:**

One hundred and thirty-five patients included: p.C634R (n = 60) and other p.C634X variants (n = 75). The p.C634R PV was associated with higher penetrance of pheochromocytoma (PHEO) (67% vs 37%, *P* < .001) and primary hyperparathyroidism (PHPT) (30% vs 15%, *P* = .036). Prevalence of MTC (87% vs 80%, *P* = .362), cutaneous lichen amyloidosis (17% vs 9% *P* = .296), and median age at initial thyroidectomy (18 vs 20 years, *P* = .479) did not differ significantly between groups. Medullary thyroid carcinoma characteristics, including stage, multifocality, extrathyroidal extension, vascular invasion, lymph node metastases, and distant metastases, were comparable. There were no significant differences in disease-free survival, overall survival, or distant metastasis-free survival for MTC. Disease status at last follow-up also was similar between p.C634R and other p.C634X PVs: no evidence of disease in 43% vs 62% (*P* = .059), biochemical evidence of disease in 25% vs 17% (*P* = .226), and structural evidence of disease in 27% vs 20% (*P* = .298).

**Conclusion:**

In this large single-center cohort, germline *RET* p.C634R was associated with a higher penetrance of PHEO and PHPT but did not increase MTC aggressiveness or worsen clinical outcomes compared with other *RET* p.C634 PVs.

Multiple endocrine neoplasia type 2 (MEN2) results from germline pathogenic variants (PVs) in the *RET* proto-oncogene, with well-established genotype–phenotype correlations that influence the age of onset and penetrance of MEN2-associated conditions (1, 2). Among these variants, *RET* p.M918T (causing MEN2B) is associated with the most severe phenotype, characterized by very early-onset medullary thyroid carcinoma (MTC) (3). In contrast, the most common PVs causing classical MEN2A occur at codon 634 within the extracellular cysteine-rich domain of the RET receptor tyrosine kinase (4). These codon 634 substitutions are typically associated with MTC arising in early childhood and are classified as high-risk mutations for which risk-reducing thyroidectomy is recommended by 5 years of age (2).

Six amino acid substitutions at codon 634 have been described (C634F/G/R/S/W/Y), with p.C634R being the most prevalent (5). Despite uniform clinical management across all codon 634 PVs, it remains unclear whether differences in amino acid substitution meaningfully influence MEN2A phenotype or MTC outcomes. Prior studies have proposed that p.C634R may confer a more aggressive MEN2A and MTC phenotype than other amino acid substitutions at codon 634 (6-8), but results have been inconsistent (9, 10). For example, a retrospective Spanish study including 199 individuals with MEN2A (173 with *RET* p.C634Y and 26 with *RET* p.C634R) reported higher penetrance of MTC, earlier age at diagnosis, increased frequency of pheochromocytoma (PHEO) and primary hyperparathyroidism (PHPT), earlier nodal and distant metastasis, and lower biochemical cure rates in p.C634R carriers (6). However, interpretation of these findings is limited by the small number of patients with p.C634R. Conversely, another retrospective cohort of 184 patients with *RET* codon 634 PVs, categorized as p.C634R, p.C634Y, and non-R/non-Y substitutions, found no differences in primary tumor diameter, nodal disease, distant metastasis, or biochemical cure among groups, although penetrance of PHEO and PHPT remained higher in p.C634R carriers (9).

Given these conflicting data (11), we aimed to compare the MEN2A phenotype and MTC outcomes in patients with germline *RET* p.C634R vs other *RET* p.C634X PVs treated at our institution to determine whether or not p.C634R is associated with a distinct or more aggressive clinical course.

## Materials and methods

### Study design and patient selection

We conducted a retrospective, single-center cohort study of patients with germline RET codon 634 PVs who were evaluated at The University of Texas MD Anderson Cancer Center (MDACC), a tertiary cancer center, and enrolled in The Medullary Thyroid Cancer Collaborative Registry (MTCCoRe). The cohort included both patients with a prior diagnosis of MEN2A who were subsequently enrolled in the registry and patients newly diagnosed during the study period. Eligible patients had been seen at MDACC at least once between May 2012, when MTCCoRe began enrolling patients, and May 2024. The data cutoff for all analyses was 1 February 2025. This study was approved by the Institutional Review Board with a waiver of informed consent (IRB# RCR04-0933).

### MTCCoRe registry description

The MTCCoRe, a multi-institutional initiative created in 2012, is designed to collect detailed demographic, clinical, pathological, and patient-reported data from individuals with MTC. The registry includes more than 1500 patients with hereditary and sporadic MTC, offering a unique opportunity to characterize the natural history of this rare disease (11). This registry includes patients with a confirmed diagnosis of MTC as well as individuals with confirmed germline PVs associated with hereditary MTC who may not yet have developed MTC. Recruitment occurs through participating institutions, where patients receive clinical care, and through direct outreach via the MTCCoRe website, patient support organizations, and other nonprofit foundations. Participant retention efforts include distribution of educational materials and newsletters and opportunities for registry participants to engage in research activities. The registry is further guided by a patient stakeholder advisory group derived from a national thyroid cancer support organization.

### Data collection and outcomes

For this study, we queried the MTCCoRe database to identify MDACC patients with germline *RET* codon 634 PVs. Extracted variables included demographic characteristics, MEN2A manifestations, MTC features, treatment history, and clinical outcomes. Disease status was determined at the time of data cutoff based on available clinical, biochemical, radiologic, and pathologic data. At last follow-up, disease status was categorized as: (1) *biochemical disease*, defined as elevation of calcitonin and/or CEA above the upper limit of normal in the absence of radiologic evidence of disease; (2) *structural disease*, defined as imaging findings suspicious for residual or recurrent disease based on radiology reports and the treating physician's assessment, with consideration of calcitonin and CEA levels when available; or (3) *no evidence of disease* (*NED*), defined as negative imaging with calcitonin and carcinoembryonic antigen (CEA) within the normal range. Most imaging was performed at MD Anderson; for imaging performed externally, outside radiology reports were used without systematic institutional re-review. Overall survival was defined as the interval from initial diagnosis of MTC to death from any cause or last follow-up, whichever came first. Disease-free survival (DFS) was defined as the time from initial surgery for MTC to first evidence of structural disease recurrence, while distant metastasis-free survival was defined as the interval from MTC diagnosis to first evidence of distant metastasis. Patients were censored at time of death or at time of last follow-up if they were alive with no evidence of structural disease recurrence or distant metastasis, respectively.

### Statistical analysis

Associations between categorical variables were assessed using Fisher's exact test. Survival outcomes including DFS, distant metastasis-free survival, and overall survival were estimated using the Kaplan–Meier method, and differences between groups were evaluated using the log-rank test. Multivariable Cox proportional hazards regression analyses were performed to evaluate factors associated with MTC outcomes. All statistical analyses were conducted using standard two-sided tests, with *P* < .05 considered statistically significant.

## Results

### Study population

A total of 427 patients with MEN2A were enrolled in the MTCCoRe and evaluated at MDACC between May 2012 and May 2024. Of these, 177 individuals harbored germline *RET* codon 634 PVs and were eligible for screening. Forty-two patients were excluded due to incomplete or missing clinical or genetic data, leaving 135 patients from 73 families for inclusion in the final analysis (Fig. 1). Among these, 60 patients (44%) carried the *RET* p.C634R variant, and 75 (56%) harbored other *RET* p.C634X substitutions.

**Figure 1:**
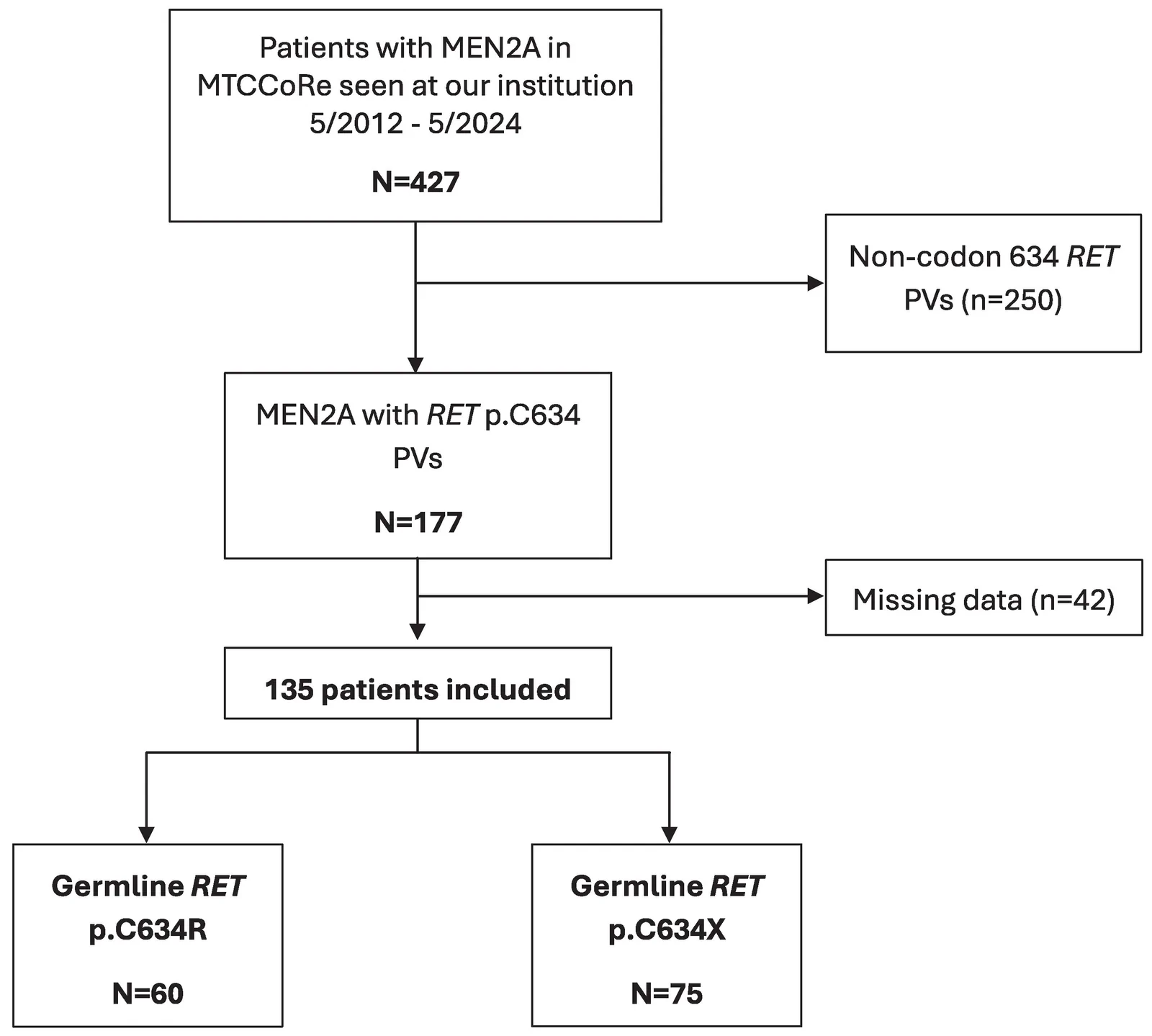
Flow diagram of study participants. Abbreviations: MEN2A, multiple endocrine neoplasia type 2A; MTCCoRE, Medullary Thyroid Cancer Collaborative Registry; PV, pathogenic variant.

### Distribution of RET codon 634 alterations

The distribution of amino acid substitutions at codon 634 is shown in Fig. 2. The p.C634R variant was the most prevalent, representing 44% of the cohort. Among the other variants, p.C634G (26%) and p.C634Y (22%) were next most common, whereas p.C634S, p.C634F, and p.C634W were each identified in fewer than 5% of patients.

**Figure 2:**
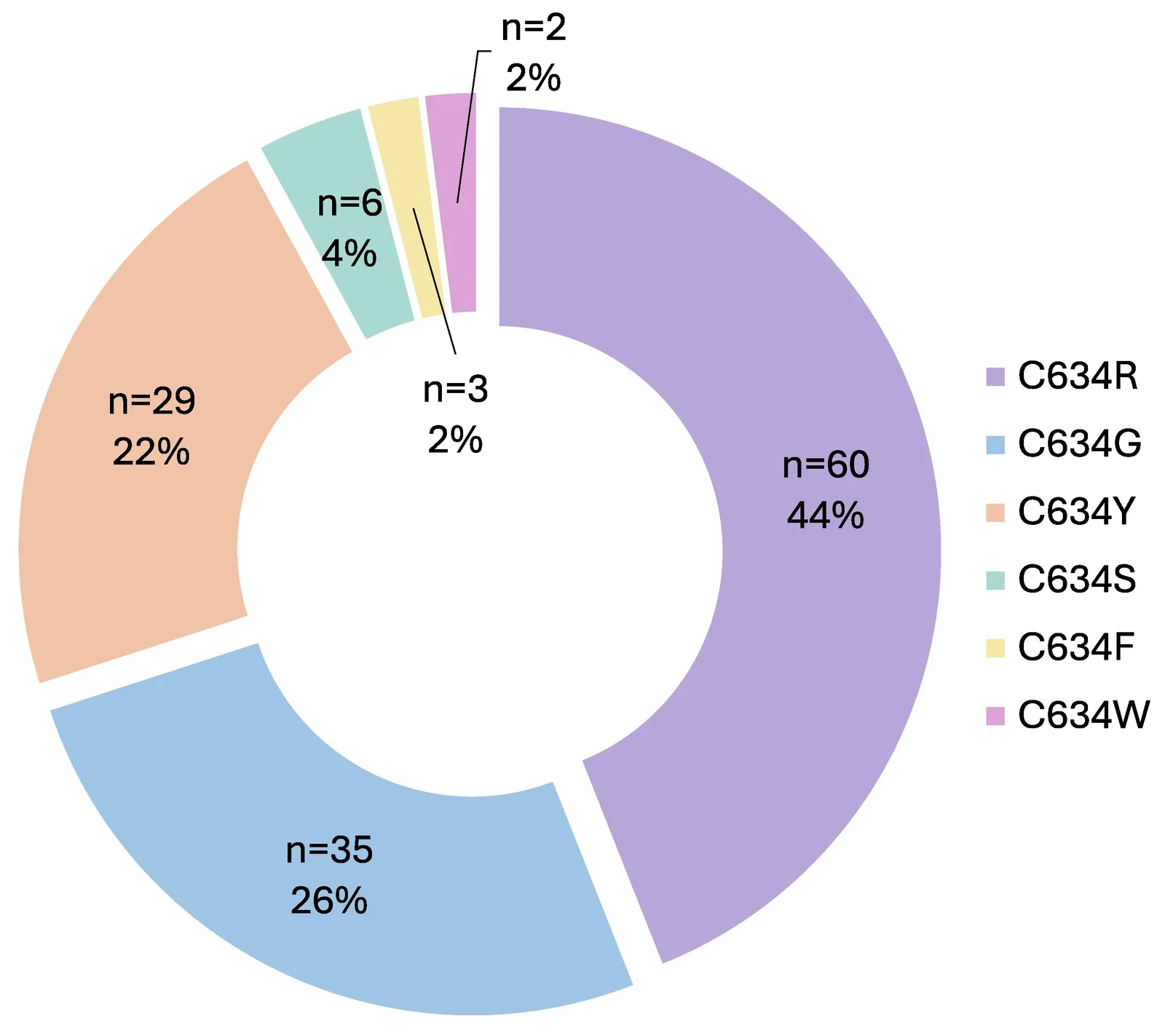
Distribution of *RET* codon 634 PVs in the study cohort (n = 135).

### Comparison of MEN2A phenotype between RET p.C634R and other p.C634X variants

Clinical characteristics of patients with *RET* p.C634R and other codon 634 PVs are summarized in Table 1. The number of patients and families was comparable between groups, with 60 patients from 37 families harboring *RET* p.C634R PVs and 75 patients from 36 families with *RET* p.C634X PVs. Both groups included nearly equal proportions of male and female patients. Median age at MEN2A diagnosis, defined as either the date of genetic confirmation or clinical diagnosis of the syndrome, was 19 years in both cohorts. Median age at histopathological MTC diagnosis (19 vs 22 years, *P* = .168) and at initial thyroidectomy (18 vs 20 years, *P* = .479) also did not differ significantly between *RET* p.C634R and other p.C634X carriers. Regarding MEN2A-associated manifestations, the prevalence of MTC (87% vs 80%, *P* = .362) and C-cell hyperplasia in patients without MTC (75% vs 80%, *P* = 1.00) was comparable. However, the p.C634R variant was associated with significantly higher penetrance of PHEO (67% vs 37%, *P* < .001) and PHPT (30% vs 15%, *P* = .036). The prevalence of cutaneous lichen amyloidosis (CLA) did not differ significantly (17% vs 9%, *P* = .296).

**Table 1:** Comparison of MEN2A phenotype and MTC characteristics between RET p.C634R and other p.C634X variants in 135 patients with MEN2A

Characteristics	*RET* p.C634R (n = 60)	Other *RET* p.C634X (n = 75)	Overall cohort (n = 135)	*P* value
Gender—no. (%)				.728
Female	31 (52)	42 (56)	73 (54)	
Male	29 (48)	33 (44)	62 (46)	
Median age at MEN2A diagnosis—years (range)	19.0 (0-47)	19.0 (0-66)	19.0 (0-66)	.893
Median age at MTC diagnosis—years (range)	19.0 (4-47)	22.0 (3-66)	20.5 (3-66)	.168
Median age at initial thyroid surgery—years (range)	18.0 (4-47)	20.0 (1-66)	19.0 (1-66)	.479
Median age at PHEO diagnosis—years (range)	31.5 (15-56)	34.0 (17-60)	32.0 (15-60)	.127
Median age at PHPT diagnosis—years (range)	27.5 (15-48)	23.0 (11-73)	24.0 (11-73)	.550
MEN2-related conditions—no. (%)				
MTC	52 (87)	60 (80)	112 (83)	.362
C-cell hyperplasia in patients without MTC	6 (75)	12 (80)	18 (78)	1.000
PHEO	40 (67)	28 (37)	68 (50)	<.001
PHPT	18 (30)	11(15)	29 (21)	.036
CLA	10 (17)	7 (9)	17 (13)	.296
MTC TNM stage—no. (%)				.589
I	22 (42)	28 (47)	50 (45)	
II	8 (15)	3 (5)	11 (10)	
III	7 (14)	8 (13)	15 (13)	
IVA	7 (14)	11 (18)	18 (16)	
IVB	0 (0)	0 (0)	0 (0)	
IVC	3 (6)	3 (5)	6 (5)	
NE	5 (9)	7 (12)	12 (11)	
Histopathological MTC characteristics				
Multifocal tumor—no. (%)	30 (81)	33 (75)	63 (77)	.597
Median tumor size—mm (range)	7.0 (1.0-70.0)	10.0 (0.5-50.0)	9.0 (0.5-70.0)	.936
Extrathyroidal extension—no. (%)	4 (13)	4 (10)	8 (11)	1.000
Lymphovascular invasion—no. (%)	7 (23)	3 (8)	10 (15)	.166
Cervical nodal metastases—no. (%)	15 (29)	20 (33)	35 (31)	.685

Abbreviations: CLA, cutaneous lichen amyloidosis; MEN2A, multiple endocrine neoplasia type 2A; MTC, medullary thyroid carcinoma; NE, nonevaluable; no, number; PHEO, pheochromocytoma; PHPT, primary hyperparathyroidism.

### Comparison of MTC characteristics and outcomes between RET p.C634R and other p.C634X variants

Medullary thyroid carcinoma clinicopathologic features, including tumor stage, multifocality, tumor size, extrathyroidal extension, vascular invasion, and cervical lymph node metastases were comparable between patients with *RET* p.C634R and those with other p.C634X variants (Table 1). Nearly all patients in both cohorts underwent thyroidectomy, with 30-45% performed as risk-reducing procedures (Table 2). The proportion of risk-reducing vs therapeutic thyroidectomies did not differ significantly between groups (*P* = .077). A small number of patients in each cohort received cytotoxic chemotherapy or neck radiation, all of which occurred before the approval of tyrosine kinase inhibitors for MTC. A few patients also underwent radiation therapy for distant metastases, with no differences observed between the two groups. Targeted systemic therapy was utilized in 11 patients overall: 6 with *RET* p.C634R and 5 with other p.C634X variants.

**Table 2:** Comparison of treatments for MTC between RET p.C634R and other p.C634X variants in 135 patients with MEN2A

	*RET* p.C634R (n = 60)	Other *RET* p.C634X (n = 75)	Overall cohort (n = 135)	*P* value
Thyroidectomy—no. (%)				1.00
Yes	59 (98)	74 (99)	133 (99)	
No	1 (2)	1 (1)	2 (1)	—
Indication for surgery—no. (%)				.077
Risk-reducing thyroidectomy	18 (30)	34 (45)	52 (39)	
Therapeutic thyroidectomy	41(68)	40 (54)	81 (60)	
Radiation therapy—no. (%)				.245
Neck radiation	6 (10)	1 (1)	7 (5)	
Radiation to distant metastases	3 (5)	5 (7)	8 (6)	
Cytotoxic chemotherapy—no. (%)	2 (3)	4 (5)	6 (4)	.692
Targeted therapy—no. (%)	6 (10)	5 (7)	11 (8)	.440

Abbreviation: no., number.

Median duration of follow-up from time of MTC diagnosis was 22.5 years (95% CI, 20.9-24.1) for the *RET* C634R cohort and 12.1 years in the other *RET* C634X cohort. Disease outcomes also were similar across groups (Table 3). Rates of locoregional recurrence (38% vs 21%, *P* = .304) and development of distant metastases at any time during the disease course (25% vs 17%, *P* = .392) did not differ significantly between *RET* p.C634R and other p.C634X carriers. The distribution of distant metastatic sites was similar, most commonly involving the liver, followed by bone and lung in decreasing frequency. Disease status at last follow-up was likewise comparable: NED in 43% vs 62% (*P* = .059), biochemical disease in 25% vs 17% (*P* = .226), and structural disease in 27% vs 20% (*P* = .298), for *RET* p.C634R and other p.C634X variants, respectively.

**Table 3:** Comparison of MTC outcomes between RET p.C634R and other p.C634X variants in 135 patients with MEN2A

	*RET* p.C634R (n = 60)	Other *RET* p.C634X (n = 75)	Overall cohort (n = 135)	*P* value
Locoregional disease recurrences—no. (%)	23 (38)	16 (21)	39(29)	.304
Distant metastases (at any time)—no. (%)	15 (25)	13 (17)	28 (21)	.392
Lung metastases	6 (10)	6 (8)	12 (9)	
Liver metastases	12 (20)	10 (13)	22 (16)	
Bone metastases	10 (17)	7 (9)	17 (13)	
Mediastinal lymphadenopathy	4 (7)	3 (4)	7 (5)	
Breast metastasis	1 (2)	0 (0)	1 (1)	
Disease status at last FU—no. (%)				.163
No evidence of disease	26 (43)	46 (62)	72 (53)	
Biochemical disease	15 (25)	13 (17)	28 (21)	
Structural disease	16 (27)	15 (20)	31 (23)	
Unknown/indeterminate	3 (5)	1 (1)	4 (3)	
Vital status at last FU—no. (%)				.817
Alive	52 (87)	66 (88)	118 (87)	
Death from any cause	8 (13)	9 (12)	17 (13)	
Death from MTC	5 (8)	3 (4)	8 (6)	

Abbreviations: no., number; FU, follow-up; MTC, medullary thyroid cancer.

After a median follow-up of 18.6 years (95% CI, 15.1-22.1), the median overall survival from the time of MTC diagnosis was 63.6 years for the entire cohort, with no significant difference between patients harboring *RET* p.C634R and other codon 634 variants (*P* = .352; Fig. 3A). Landmark survival estimates were also similar between genotype groups. Five-year MTC-specific survival was 98.0% in both the C634R and other C634 groups. At 10 years, MTC-specific survival was 98.0% for C634R and 95.6% for other C634 variants, and at 20 years, survival was 95.6% and 92.5%, respectively. Similarly, structural DFS and distant metastasis-free survival did not differ between the two groups (Fig. 3B and 3C). Moreover, on multivariable Cox proportional hazards analysis, RET C634R genotype was not independently associated with DFS or MTC-specific mortality after adjustment for relevant clinical and treatment-related factors including sex, age at MTC diagnosis, RET genotype (C634R vs other C634 variants), stage at diagnosis, indication for thyroidectomy (risk-reducing vs therapeutic), lymph node dissection, neck radiation therapy, and systemic therapy (Table S1 (12)). Older age at MTC diagnosis (HR, 1.08; 95% CI, 1.01-1.16) and receipt of systemic therapy (HR, 3.85; 95% CI, 1.06-14.04) were the only factors associated with higher MTC-specific mortality, while only receipt of systemic therapy (HR, 4.36; 95% CI, 1.59-11.94) were associated with worse DFS.

**Figure 3:**
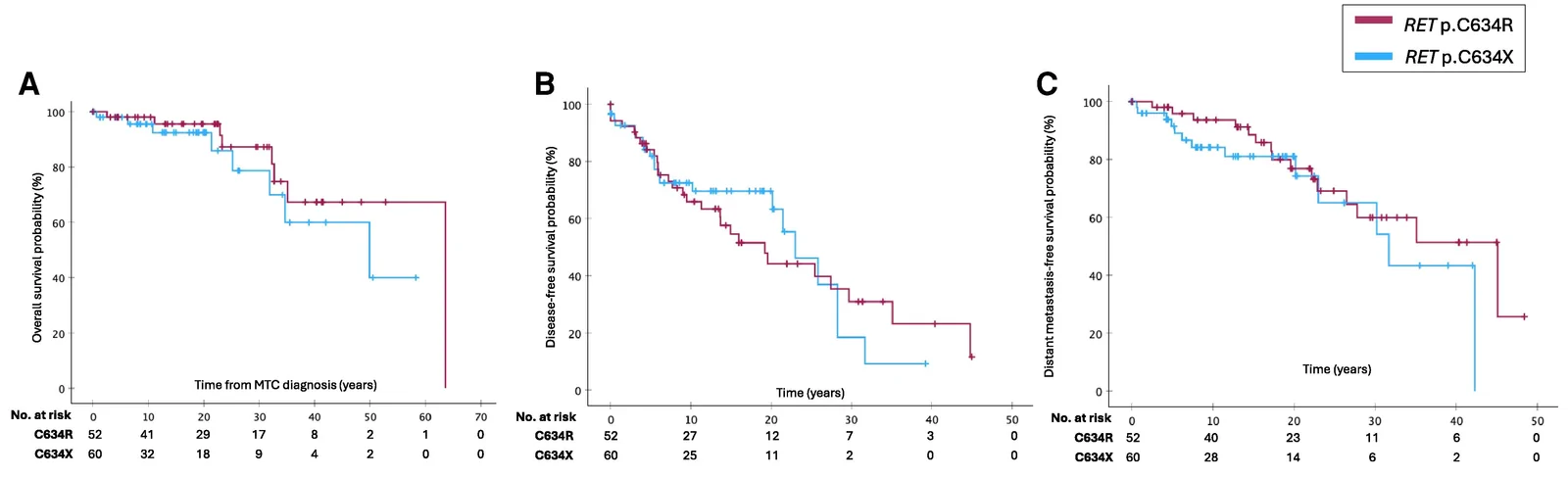
Kaplan–Meier plots comparing (A) overall survival (*P* = .353) (B) structural disease-free survival (*P* = .835), and (C) distant metastasis-free survival (*P* = .343) between *RET* p.C634R and other p.C634X variants in 135 patients with MEN2A.

At the time of data cutoff, 17 patients had died, at a median of 24.3 (range, 0.6-63.6) years from diagnosis. Of these, 8 deaths (47%) were related to MTC, 5 in patients with a *RET* p.C634R PV and 3 in those with other p.C634X variants, and occurred at a median age of 41 years (range, 29-68).

## Discussion

Although genotype–phenotype correlations between specific *RET* PVs and the clinical manifestations of MEN2 are well established, it remains uncertain whether different amino acid substitutions at the same codon meaningfully influence disease presentation or outcomes. In this large single-center cohort of patients with MEN2A and germline *RET* codon 634 PV, we found that the *RET* p.C634R variant was associated with a higher penetrance of PHEO and PHPT compared with other p.C634X substitutions, but we did not detect evidence of a more aggressive MTC phenotype or worse oncologic outcomes among p.C634R carriers. MTC stage at diagnosis, pathologic features, recurrence rates, development of distant metastases, and survival outcomes were comparable between patients with *RET* p.C634R and those with other codon 634 variants. Importantly, C634R genotype remained unassociated with DFS or MTC-specific mortality in multivariable analyses adjusting for clinical and treatment-related factors, including age at diagnosis, indication for thyroidectomy, stage, and subsequent therapies. While these results align with some prior studies indicating that genotype does not meaningfully predict MTC outcomes after diagnosis (13, 14), they contradict others suggesting that p.C634R is associated with more aggressive MTC features (6-8).

Our findings contribute to the clinically relevant debate regarding phenotypic variability among *RET* codon 634 PVs, which remain uniformly classified as high-risk in current American Thyroid Association guidelines (2). Prior studies addressing this question have yielded inconsistent results. An early Brazilian cohort (7) of 47 patients with MEN2A and *RET* codon 634 PVs reported a more aggressive MTC course in *RET* p.C634R carriers compared with p.C634Y and p.C634W, including earlier lymph node and distant metastases, and more M1 disease at diagnosis; however, this study largely reflected the natural history of disease prior to routine genetic screening and risk-reducing thyroidectomy, potentially exaggerating genotype-specific differences. In contrast, the multicenter EUROMEN study demonstrated age-related progression from C-cell hyperplasia to MTC and nodal disease but found that the specific amino acid substitution at codon 634 did not influence early malignant progression, supporting codon-level rather than amino acid-specific risk stratification (10). Similarly, a large German surgical series by Machens and Dralle (9) reported higher penetrance of MEN2A-associated manifestations in p.C634R carriers, including PHEO and PHPT, but observed no differences in MTC-related outcomes such as tumor size, nodal involvement, distant metastases, or biochemical cure, leading the authors to conclude that these differences did not warrant differential clinical management.

In contrast, a Spanish national registry study by Valdés et al (6) found that the germline *RET* p.C634R PV was associated with earlier MTC onset, earlier development of nodal and distant metastases, and worse disease-specific outcomes compared with p.C634Y. However, interpretation of these findings is limited by the relatively small number of p.C634R carriers included in the study (26/199). More recently, a large multicenter cohort from China and Brazil (8) including 317 patients with germline *RET* codon 634 PVs from 138 families, reported increased MTC aggressiveness among *RET* p.C634R carriers, with higher rates of distant metastases and worse disease-specific survival. Taken together, these studies raise the possibility that amino acid-specific differences at codon 634 may influence the natural history of MTC, even though such an association was not detected in our cohort. The reasons for these discrepant findings remain uncertain but may reflect differences in study populations, including geographic and ethnic variation, referral and ascertainment patterns, duration of follow-up, and access to early genetic testing and risk-reducing thyroidectomy. Notably, patients in our cohort were diagnosed with MEN2 and underwent initial thyroid surgery at a median age approximately 10 years younger than those in the China–Brazil cohort. Earlier identification and intervention may have limited progression to advanced MTC and thereby attenuated genotype–phenotype differences that might be more apparent in cohorts diagnosed later in the natural history of disease. This hypothesis remains speculative and cannot be established from the present retrospective analysis.

Our study has several limitations that merit consideration. First, the retrospective, single-center design introduces the potential for referral, ascertainment, and selection bias. Although our cohort does not appear to be predominantly enriched for advanced disease, with 55% of patients with MTC presenting with stages I-II disease and 39% of the overall cohort undergoing risk-reducing thyroidectomy, patients evaluated at a tertiary cancer center and enrolled in an institutional registry may not be representative of the broader MEN2A population. As discussed above, differences in referral and ascertainment may have influenced the observed genotype–phenotype associations and limit the generalizability of our findings. Additionally, imaging was not centrally adjudicated, and outside imaging studies were not systematically re-reviewed at our institution, which may have introduced variability in the classification of structural disease, particularly for indeterminate findings. Follow-up was substantially longer in the RET C634R cohort than in the other RET C634X cohort (median 22.5 vs 12.1 years), which could influence comparisons of long-term outcomes. Although MTC-specific survival estimates were similar between groups at fixed 5-, 10-, and 20-year time points, the shorter follow-up in the other RET C634X cohort remains an important limitation and may have reduced the ability to detect very late recurrences, distant metastases, or MTC-related deaths.

Furthermore, despite the relatively large size and long follow-up of our cohort, the absence of statistically significant differences in MTC outcomes cannot definitively establish equivalence between p.C634R and other codon 634 variants. The study may have had limited power to detect modest differences in relatively infrequent outcomes such as distant metastases and MTC-specific mortality. Subgroup analyses, particularly for less common manifestations such as CLA, were also limited by small numbers. Additionally, patients enrolled in MTCCoRe may not fully reflect the broader MEN2A population. As previously reported (11), the registry includes a younger patient population, fewer racial and ethnic minority patients, and a higher proportion of individuals receiving systemic therapies compared with state and national population-based datasets, which may limit generalizability. In addition, because this analysis was limited to patients enrolled in MTCCoRe, some individuals evaluated at our institution, including pediatric patients who may not have been captured in the registry, were not included in this cohort. These limitations and the discordant findings across existing studies underscore the critical need for collaborative, multi-institutional, and multinational registries to better characterize the full phenotypic spectrum and outcomes of patients with MEN2.

In conclusion, in this large single-center MEN2A cohort with long-term follow-up, the *RET* p.C634R pathogenic variant was associated with higher penetrance of PHEO and PHPT but we did not observe greater MTC aggressiveness or worse oncologic outcomes compared with other *RET* codon 634 substitutions. Together with the conflicting findings of prior studies, our results highlight that the clinical significance of amino acid-specific differences at codon 634 for MTC remains unsettled. Further large, multinational studies are needed to clarify whether individual codon 634 substitutions independently influence MTC outcomes and to identify factors that may account for differences observed across cohorts.

## Data Availability

Some or all datasets generated during and/or analyzed during the current study are not publicly available but are available from the corresponding author on reasonable request.
